# Bibliometric and visualization analysis of macrophages associated with osteoarthritis from 1991 to 2021

**DOI:** 10.3389/fimmu.2022.1013498

**Published:** 2022-10-04

**Authors:** Zhen Yang, Jianjing Lin, Hui Li, Zihao He, Kai Wang, Liandi Lei, Hao Li, Dan Xing, Jianhao Lin

**Affiliations:** ^1^ Arthritis Clinical and Research Center, Peking University People’s Hospital, Beijing, China; ^2^ Arthritis Institute, Peking University, Beijing, China; ^3^ Department of Sports Medicine and Rehabilitation, Peking University Shenzhen Hospital, Shenzhen, China; ^4^ Center of Medical and Health Analysis, Peking University, Beijing, China; ^5^ School of Medicine, Nankai University, Tianjin, China

**Keywords:** osteoarthritis, macrophages, bibliometric, CiteSpace, VOSviewer

## Abstract

**Background:**

Macrophages significantly contributes to symptomology and structural progression of osteoarthritis (OA) and raise increasing attention in the relative research field. Recent studies have shown that tremendous progress has been made in the research of macrophages associated with osteoarthritis. However, a comprehensive bibliometric analysis is lacking in this research field. This study aimed to introduce the research status as well as hotspots and explore the field of macrophages research in OA from a bibliometric perspective.

**Methods:**

This study collected 1481 records of macrophages associated with osteoarthritis from 1991 to 2021 in the web of science core collection (WoSCC) database. CiteSpace, VOSviewer, and R package “bibliometrix” software were used to analyze regions, institutions, journals, authors, and keywords to predict the latest trends in macrophages associated with osteoarthritis research.

**Results:**

The number of publications related to macrophages associated with osteoarthritis is increasing annually. China and the USA, contributing more than 44% of publications, were the main drivers for research in this field. League of European Research Universities was the most active institution and contributed the most publications. *Arthritis and Rheumatism* is the most popular journal in this field with the largest publications, while *Osteoarthritis and Cartilage* is the most co-cited journal. Koch AE was the most prolific writer, while Bondeson J was the most commonly co-cited author. “Rheumatology”, “Orthopedics”, and “Immunology” were the most widely well-represented research areas of OA associated macrophages. “Rheumatoid arthritis research”, “clinical symptoms”, “regeneration research”, “mechanism research”, “pathological features”, and “surgery research” are the primary keywords clusters in this field.

**Conclusion:**

This is the first bibliometric study comprehensively mapped out the knowledge structure and development trends in the research field of macrophages associated with osteoarthritis in recent 30 years. The results comprehensively summarize and identify the research frontiers which will provide a reference for scholars studying macrophages associated with osteoarthritis.

## Introduction

Osteoarthritis (OA) remains the most common form of arthritic disease which affects the whole joint. By 2030, there would be 35% of people in the general population suffering from OA, and it is predicted to be the single greatest cause of disability (1). In the USA, over 27 million OA patients are estimated to suffer from this disease, and caused tremendous social and economic burdens (2). It is now accepted that some risk factors such as genetic predisposition, obesity, aging, and joint trauma plays a major role in OA development (3). Despite improved pain alleviation through the development of treatment therapies, the joint function restoration and damaged cartilage repair for OA patients is still lacking promising advances (4). Recently, OA has been defined as a low-degrade inflammatory disease that involving cartilage loss, synovitis, subchondral bone remodeling, osteophyte formation and meniscus and ligament changes (5). Therefore, it is urgent to elucidate the pathophysiological basis of inflammation and tissue damage repair processes of OA to benefit the advances of prognosis and therapeutics of OA diseases.

In recent years, the role of macrophage-mediated inflammation in the pathogenesis of OA has gained wide attention. Currently, the role of synovial inflammation in the OA progression still remains to be determined. It has been demonstrated that multiple factors act as danger-associated molecular patterns (DAMPs) that result in macrophage activation can initiate synovial inflammation during OA. One possible theory is that, exogenous pathogen-associated molecular patterns (PAMPs) and endogenous DAMPs selectively activate surface pattern recognition receptors (PRRs) on macrophages, subsequently induce inflammatory cytokines and chemokines secretion (6). Another primary activation way refers to inflammasome mediated pathways, such as the NLR pyrin domain containing 3 (NLRP3) inflammasome. NLRP3, belongs to a member of NLR family, was proved to recognize different DAMPs to form NLRP3 inflammasome in the cytosol and initiate inflammations (7). As such, macrophages could serve as a possible treatment target in OA. For example, the clearance of macrophages by anti-CD14-conjugated magnetic beads successfully reduce production of IL-1 and TNF-α (8). Moreover, as a kind of plastic cells, macrophages are classified as classically activated M1 and alternatively activated M2 macrophages (9). The macrophage subtypes can be generated *in vitro*, as interferon (IFN)-γ/lipopolysaccharide (LPS) can induce M1 subtype formation while M2 macrophages can be generated by exposing M0 macrophages to interleukin (IL)-4/IL-13 (10, 11). Compared to pro-inflammatory M1 macrophages, M2 macrophages are known as immunomodulatory macrophages and contribute to tissue repair and regeneration (12, 13). This information indicates the significance of regulating macrophage polarization in alleviating OA progression. For instance, a canine OA model treated with intra-articular injections of recombinant human IL-1ra which refer to M2 marker presented an reduction of osteophytes formation and cartilage loss (14). However, the imbalance between M1 and M2 macrophages requires further investigations and new advances of macrophage reprogramming may yield significance for prevent OA. Despite the increasing interests on the topic of OA associated macrophages, comprehensive and meaningful analysis of publication trends of this research area remains highly insufficient and requires to be summarized urgently.

Recently, bibliometric analysis has been widely adopted to analyze massive scientific research data and identify developing trends (15). Importantly, it can summarize publication evolution, predict research hotspots, and further evaluate frontiers in specific fields though a citation network (16–18). As far as we know, although related academic researchers have published bibliometric studies of stem cells in OA (19), no similar analysis about macrophage in OA have as yet been reported. Notably, several bibliometric tools such as CiteSpace, VOSviewer, R package “bibliometrix” have been applied to visualize the specific medical literature analysis fields (20–22). Therefore, in the present study, we used bibliometric statistics to fill this knowledge gap. This paper comprehensively analyzed the literatures related to OA associated macrophages and performed visualization analysis over the last three decades (from 1991 to 2021) to identify its significant features and predict future research directions.

## Materials and methods

### Data source and search strategy

Web of science core collection (WoSCC) database originating from Clarivate Analytics was considered one of the most authoritative and comprehensive database platforms which contains more than 12000 international academic journals (23). Therefore, we selected it to obtain global academic information for bibliometric analysis according to previous studies (24–26). All the published literatures were extracted from WOS and the date of the search were from 1 January 1991 to 31 December 2021. In present study, the search terms were as follows: theme = osteoarthritis or degenerative arthritis *AND theme = macrophage or macrophages or histocyte or histocytes AND publishing year = (1991–2021) AND Document types = (ARTICLE OR REVIEW) AND Language = (English). The detailed information of certain countries of regions in the WoSCC was refined by indexing country/region when search. Additionally, all valid data of literatures, including publishing year, title, author names, nationalities, affiliations, abstract, keywords, and name of journals were saved in the format of download.txt files from WoSCC database and subsequently imported into Excel 2021. Coauthors (YZ and LJJ) independently searched and extracted all data from these literatures. Any disagreement was resolved by consulting with experts to reach the final consensus. Finally, all the coauthors separately cleaned and analyzed the data with Origin 2021 and GraphPad Prism 8.

### Bibliometric analysis and visualization

As we know, the intrinsic function of WoSCC was to explore the basic features of eligible literatures. Therefore, the number of literatures and corresponding citations were reflected. The relative research interest (RRI) was deemed as the number of publications in a certain field by all field literatures per year. The world map was acquired by R software including python + numpy + scipy + matplotlib. The time curve of publications was drawn according to previous article (19). The H-index, which refers to a scholar who has published H papers and they have been cited at least H times, was defined to measure the impact of scientific research (27). We chose the VOSviewer (Leiden University, Leiden, The Netherlands) software to construct and visualize bibliometric networks of the publications in our present study. And the VOSviewer was performed for analyzing the bibliographic coupling, co-citation, and co-occurrence analyses in detail. In addition, we choose R package “bibliometrix” software to visualize publications production among countries, map the international collaboration between countries, and visualize a three-field plot analysis. Moreover, CiteSpace (6.1. R2) which was developed by Professor Chen C, was used to construct dual-map overlay for journals, cluster analysis of co-cited keywords, and detection of references and keywords with intense citation bursts.

## Results

### Overall performance of global literatures

**Figure 1:**
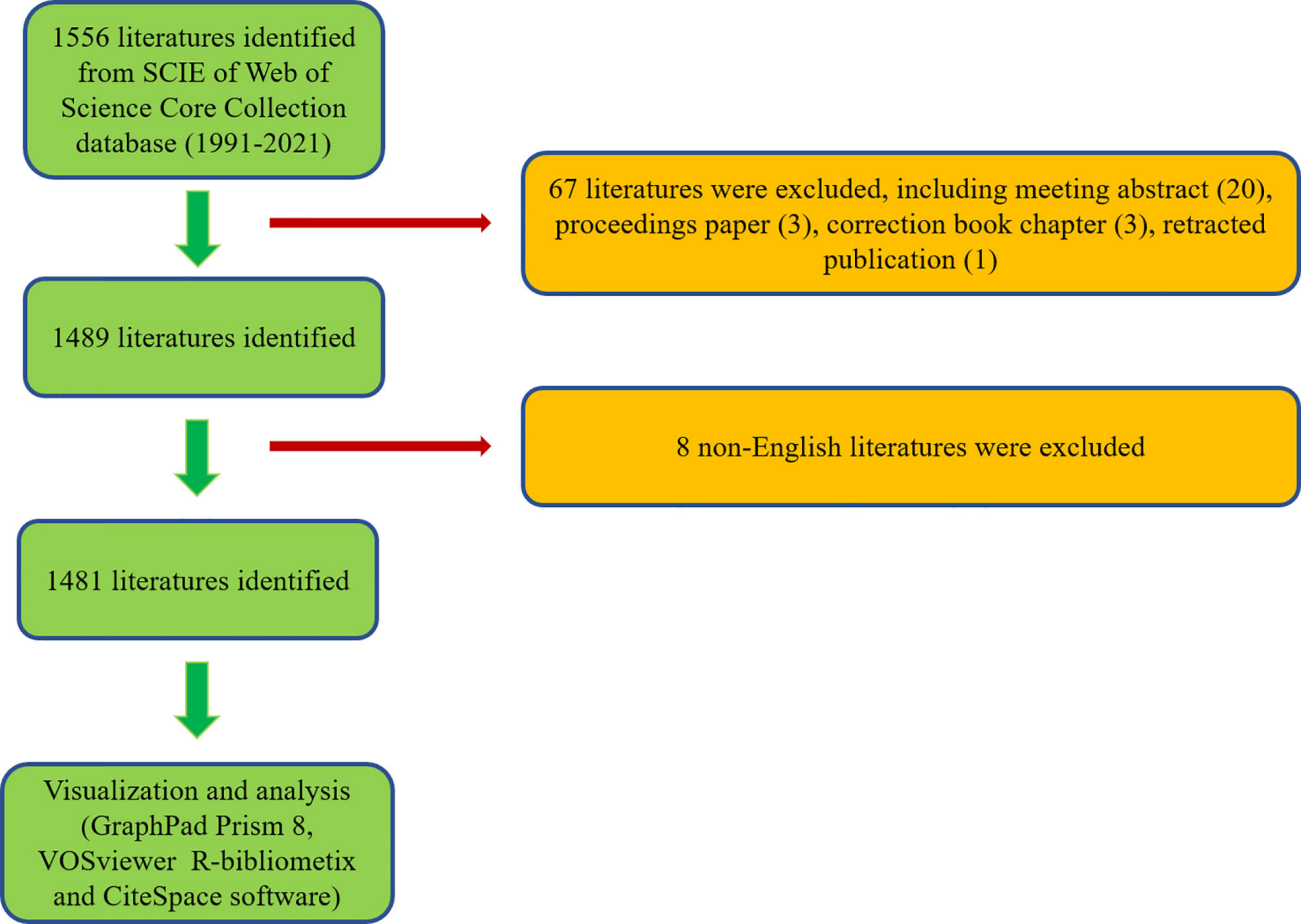
Flowchart of the screening process.

**Figure 2:**
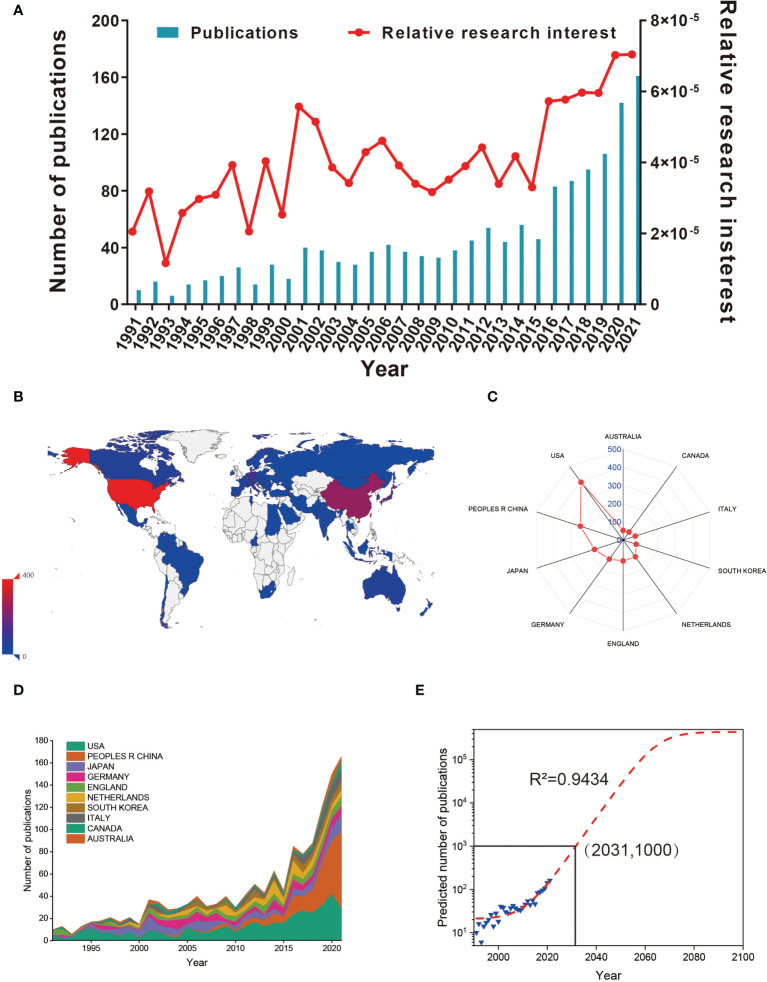
**(A)** The global number (blue bars) and relative research interests (red curve) of publications related to macrophages associated with osteoarthritis. **(B)** Distribution of macrophages associated with osteoarthritis research in world map. **(C)** The sum of publications related to macrophages associated with osteoarthritis from the top 10 countries and regions. **(D)** The annual number of publications in the top 10 most productive countries from 1991 to 2021. **(E)** Model fitting curves of global trends in publications related to macrophages associated with osteoarthritis per year (R^2 =^ 0.9434, (2031,1000) indicates that the total publications will up to 1000 in year of 2031).

According to the search criteria, a total of 1556 literatures were collected from the year of 1991 to 2021. Subsequently, 1489 of literatures were identified by excluding the meeting abstract (20), proceedings papers (3), correction book chapter (3), and retracted publication (1). Finally, 1481 literatures were identified by excluding 8 non-English literatures (**Figure 1**). As shown in **Figure 2A**, the trend of global literatures was increasing steadily year by year. The number of literatures increased from 10 (1991) to 161 (2021). The most research was published in 2021 (161, 11.14%) (**Figure 2A**). In addition, the relative interest in this field has also increased over the past few years (**Figure 2A**).

In total, 65 countries/regions have made contributions in literatures in this field. As shown in **Figures 2B, C**, the USA published the most papers (394, 29.266%), followed by China (247, 17.093%), Japan (166, 11.488%), Germany (129, 8.927%) and England (115, 7.958%). It is shown in **Figure 2D** that the annual number of publications of top 10 countries/regions rose from 10 (0.705%) in 1991 to 166 (11.707%) in 2021. Before 2019, the annual number of publications of the USA and Japan increased faster than that of China. For predicting the future global literatures trend, a logistic regression model was performed to create a time curve of the number of literatures. **Figure 2E** illustrates the fitting curve of the annual publication trend and the correction coefficient R2 is 0.9434. The predicted number of publications will be was estimated to 1000 in the year of 2031. Overall, these results indicating that the research on macrophages associated with osteoarthritis has attracted increasing researchers’ focus and reached a staged of rapid development.

### Analysis of countries

**Figure 3:**
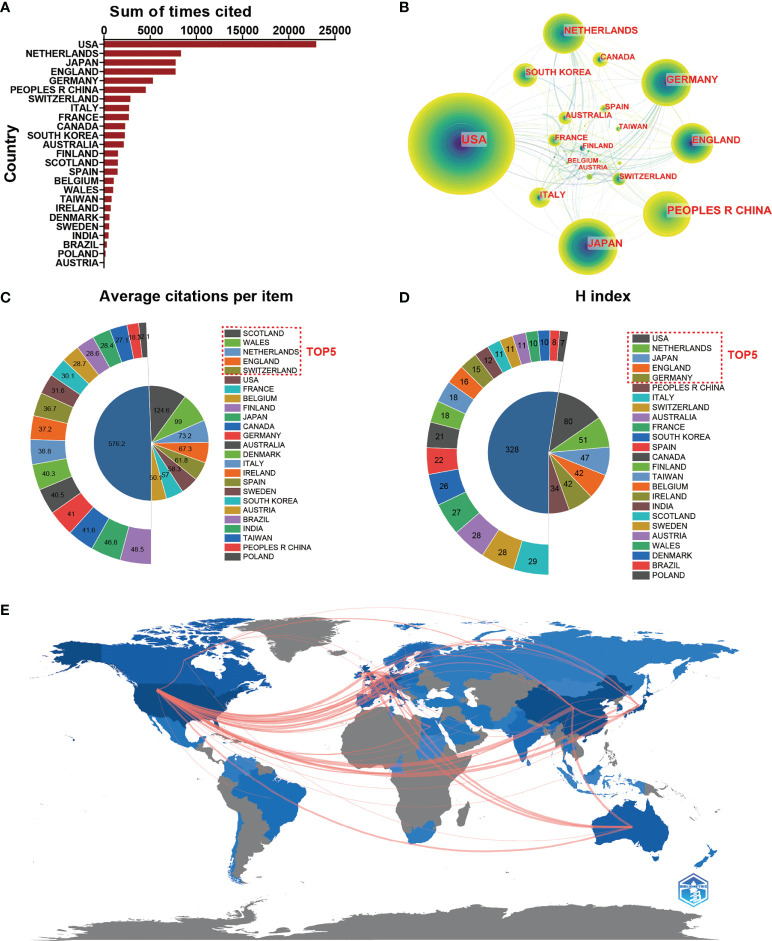
**(A)** The top 25 countries/regions of total citations related to macrophages associated with osteoarthritis. **(B)** Country/regional collaboration analysis. **(C)** The top 25 countries/regions of the average citations per publication related to macrophages associated with osteoarthritis. **(D)** The top 25 countries/regions of the publication H-index related to macrophages associated with osteoarthritis. **(E)** The geographical network map of macrophages associated with osteoarthritis.

As we can see from **Figure 3A**, publications from the USA had the highest total citation frequencies (22978). Netherlands ranked second in total citation frequencies (8340), followed by Japan (7760), England (7744) and Germany (5291). Regarding the global collaboration network analysis, the **Figure 3B** showed that the USA exhibited the highest output volume and worked closely with Netherland, South Korea, and France. From the **Figure 3E**, we can figure out that the network diagram of cooperation mainly exists in North America, West Europe, and East Asia. In terms of every citation frequency, publications from Scotland had the highest average citation frequencies (124.58). Wales ranked second in average citation frequency (99), prior to the Netherlands (73.16), England (67.34) and Switzerland (61.76) (**Figure 3C**). Additionally, the USA (80) dominated in this field in the relative publications of H-index, followed by Netherlands (51), Japan (47), England (42) and Germany (42) (**Figure 3D**).

### Analysis of institutions and authors

**Figure 4:**
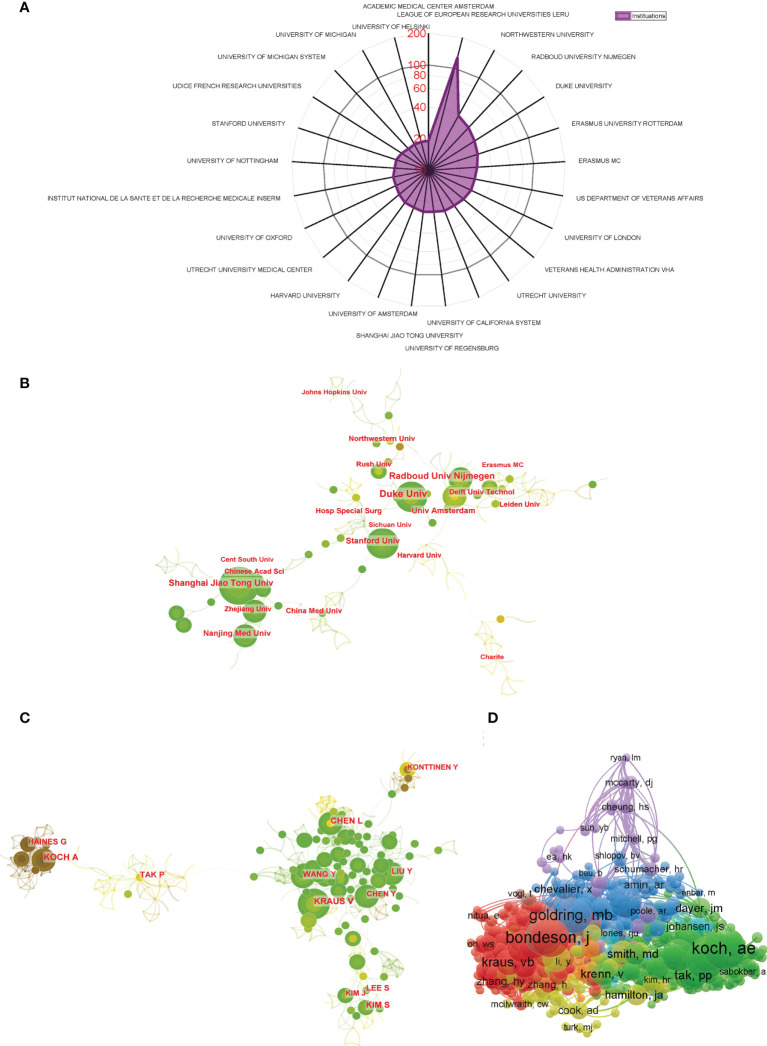
**(A)** The top 25 institutions with most publications related to macrophages associated with osteoarthritis. **(B)** Institutional collaboration analysis. **(C)** Author collaboration analysis. **(D)** Network visualization diagram of the co-cited authors of the Publications.

Regarding publication ranking, the top 25 contributive institutions were listed in **Figure 4A**. The first was League of European Research Universities (127 publications), followed by Northwestern University (39 publications), and Radboud University Nijmegen ranked third (36 publications). **Figure 4B** exhibits the network diagram of collaboration between institutions, which shows that that there is strong cooperation relationship between institutions such as Shanghai Jiao Tong University, Zhejiang University, and Nanjing Medical University in China and Duke University, Stanford University, and Harvard University in the USA.

The top 10 authors contributed a total of 217 publications, which accounted for approximately 15% of all publications in this field. Koch AE published the most studies, with 29 publications, followed by Haines GK with 26 publications and Tak PP with 23 publications (**Table 1**). CiteSpace visualizes the network between authors, as shown in **Figure 4C**. Authors from the same country collaborate more frequently with strong connection. However, the connections between authors from different countries are still inadequate. The co-citation analysis considered the relatedness of the items based on the numbers they were co-cited. A total of 871 authors with a minimum of 10 documents were analyzed using VOSviewer (**Figure 4D**). The top 5 authors with largest total link strength were as follows: Bondeson J (total link strength =5889 times), Blom AB (total link strength = 5513 times), Goldring MB (total link strength = 4692 times), Scanzello CR (total link strength = 4543 times), and Koch AE (total link strength = 4359 times).

**Table 1:** The top 10 authors with the most publications on macrophages associated with osteoarthritis.

Rank	High Published Authors	Country	Article counts	Percentage %
1	Koch AE	USA	29	2.007
2	Haines GK	USA	26	1.799
3	Tak PP	Netherlands	23	1.592
4	Van Den Berg WB	Netherlands	22	1.522
5	Kraus VB	USA	21	1.453
6	Pope RM	USA	20	1.384
7	Van Der Kraan PM	Netherlands	20	1.384
8	Straub RH	Germany	19	1.315
9	Van Lent PLEM	Netherlands	19	1.315
10	Van Osch GJVM	Netherlands	18	1.246

### Analysis of journals and research areas

**Figure 5:**
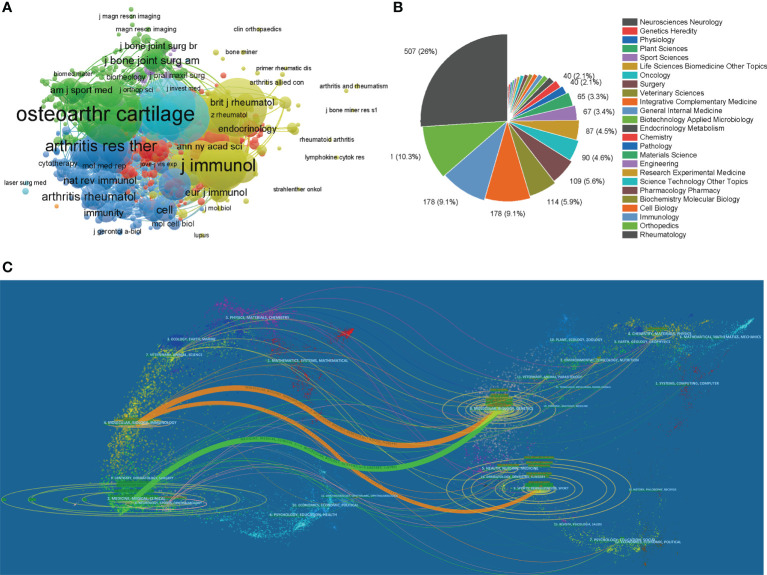
**(A)** Network map of journals that were co-cited in more than 20 citations. **(B)** Mapping of the top 25 research directions related to macrophages associated with osteoarthritis. **(C)** The dual-map overlay of journals related to macrophages associated with osteoarthritis.

**Table 2** lists the top 10 productive journals involved in this study. The journal *Arthritis and Rheumatism* (impact factor = 8.955, 2021) published the most with 98 publications. There were 92 publications in *Osteoarthritis and Cartilage* (IF = 7.507, 2021), 77 publications in *Arthritis Research Therapy* (IF = 5.606, 2021), 47 publications in *Journal of Rheumatology* (IF = 5.346, 2021) and 45 articles in *Annals of the Rheumatic Diseases* (IF = 27.973, 2021). The names of journals of co-citation analysis were performed using VOSviewer, and the journal with a minimum number of citations over 10 was defined. As plotted in **Figure 5A**, 824 journals were shown in the total link strength. The top 5 journals with best total link strength were as follows: *Osteoarthritis and Cartilage* (total link strength = 184826 times), *Arthritis and Rheumatism* (total link strength =152813 times), *Annals of the Rheumatic Diseases* (total link strength = 135410 times), *Journal of Immunology* (total link strength = 105307 times), and *Arthritis Research Therapy* (total link strength = 93494 times).

**Table 2:** The top 10 productive journals related to macrophages associated with osteoarthritis.

Rank	Journal	Article counts	Percentage%	IF
1	Arthritis and Rheumatism	98	6.773	8.955
2	Osteoarthritis and Cartilage	92	6.358	7.507
3	Arthritis Research Therapy	77	5.321	5.606
4	Journal of Rheumatology	47	3.248	5.346
5	Annals of the Rheumatic Diseases	45	3.110	27.973
6	Journal of Orthopedic Research	33	2.281	2.728
7	Arthritis Rheumatology	27	1.866	15.483
8	Plos One	25	1.728	3.752
7	Scientific Reports	25	1.728	4.996
10	Journal of Immunology	22	1.520	5.426

We performed a visual analysis of the research orientations using VOSviewer (**Figure 5B**), which is also summarized in **Table 3**. In details, the most prevalent research fields were rheumatology, orthopedics, immunology, cell biology, and biochemistry molecular biology. The spline wave from left to right describes the citation association, which is represented by the colored path. The **Figure 5C** depicted three primary citation paths marked in orange and green. The two primary paths showed that documents published in molecular/biology/genetics were primarily cited by researchers published in molecular/biology/immunology and medicine/medical/clinical journals, while the third path showed that documents published in sports/rehabilitation/sport was primarily cited by researchers published in molecular/biology/immunology.

**Table 3:** The top 10 well-represented research areas related to macrophages associated with osteoarthritis.

Rank	Research Areas	Records	Percentage%
1	Rheumatology	508	35.107
2	Orthopedics	201	13.891
3	Immunology	178	12.301
4	Cell Biology	155	10.712
5	Biochemistry Molecular Biology	107	7.395
6	Pharmacology Pharmacy	102	7.049
7	Medicine Research Experimental	87	6.012
8	Multidisciplinary Sciences	73	5.045
9	Engineering Biomedical	63	4.354
10	Materials Science Biomaterials	56	3.870

### Citation and co-citation analysis

**Figure 6:**
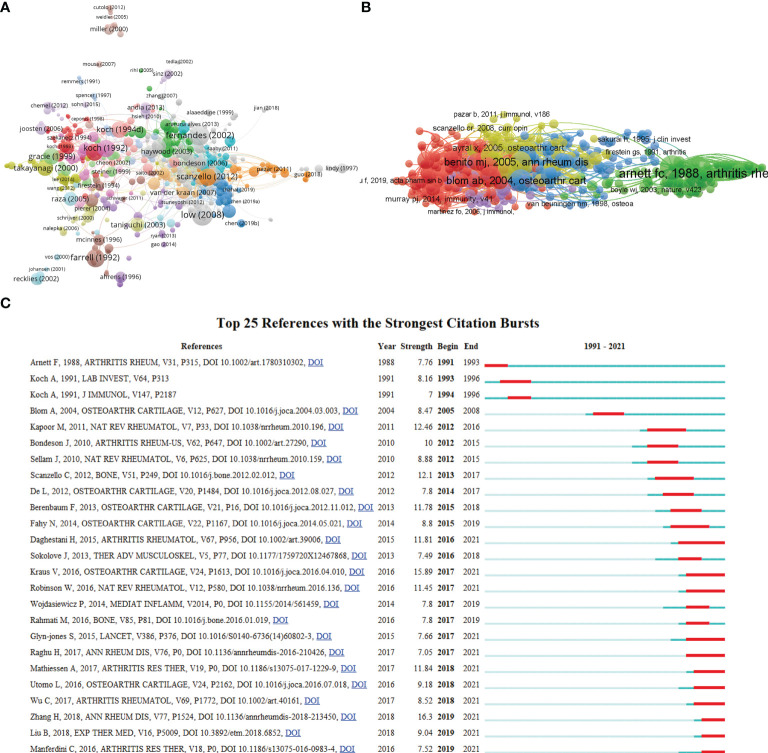
**(A)** Network map of citation analysis of documents with more than 25 citations. **(B)** Network map of co-citation analysis of references. **(C)** Top 25 references with strongest citation bursts of publications related to macrophages associated with osteoarthritis.

A total of 674 articles in this field have more than 25 citations (**Figure 6A**). The top 10 most cited documents are shown in **Table 4**. There were 878 citations for “Discovery and development of folic-acid-based receptor targeting for Imaging and therapy of cancer and inflammatory diseases”, followed by “The role of cytokines in osteoarthritis pathophysiology”, with 784 citations. The third-ranked article with the largest number of citations was “Increased Concentrations of Nitrite in Synovial-Fluid and Serum Samples Suggest Increased Nitric-Oxide Synthesis in Rheumatic Diseases”, with 624 citations.

**Table 4:** The top 10 documents with the most citations in the field of macrophages associated with osteoarthritis.

Rank	Title	Corresponding Author	Journal	IF	Publication year	Total citations
1	Discovery and development of folic-acid-based receptor targeting for Imaging and therapy of cancer and inflammatory diseases	Doorneweerd, DD	Accounts of Chemical Research	24.466	2008	878
2	The role of cytokines in osteoarthritis pathophysiology	Pelletier, JP	Biorheology	1.615	2002	784
3	Increased Concentrations of Nitrite in Synovial-Fluid and Serum Samples Suggest Increased Nitric-Oxide Synthesis in Rheumatic Diseases	Moncada, S	Annals of The Rheumatic Diseases	27.973	1992	624
4	The role of synovitis in osteoarthritis pathogenesis	Goldring, SR	Bone	4.626	2012	595
5	Enhanced Production of Monocyte Chemoattractant Protein-1 In Rheumatoid-Arthritis	Strieter, RM	Journal of Clinical Investigation	19.456	1992	579
6	Localization of Tumor-Necrosis-Factor-Alpha in Synovial Tissues and At the Cartilage Pannus Junction in Patients with Rheumatoid-Arthritis	Maini, RN	Arthritis and Rheumatism	8.955	1991	537
7	A proinflammatory role for IL-18 in rheumatoid arthritis	McInnes, IB	Journal of Clinical Investigation	19.456	1999	531
8	A clinical perspective of IL-1 beta as the gatekeeper of inflammation	Dinarello, CA	European Journal of Immunology	6.688	2011	520
9	Vascular Endothelial Growth-Factor - A Cytokine Modulating Endothelial Function in Rheumatoid-Arthritis	Ferrara, N	Journal of Immunology	5.426	1994	518
10	Involvement of receptor activator of nuclear factor kappa B ligand/osteoclast differentiation factor in osteoclastogenesis from synoviocytes in rheumatoid arthritis	Tanaka, S	Arthritis and Rheumatism	8.955	2000	483

Moreover, co-cited references were analyzed by VOSviewer (**Figure 6B**) to show the most influential literature. In addition, citation burst is a valuable indicator that reflects the references of interest to researchers in a particular domain in a period (28). In our study, the top 25 references with the strongest citation bursts were identified by CiteSpace and presented in **Figure 6C**, among which the citation burst for duration of references. The article titled “Synovial macrophage M1 polarisation exacerbates experimental osteoarthritis partially through R-spondin-2”, published in 2018, ranked first (strength = 16.3). Meanwhile, the citation bursts of articles published by Daghestani H lasted from 2016 to 2021.

### Analysis of keywords and hotspots

**Figure 7:**
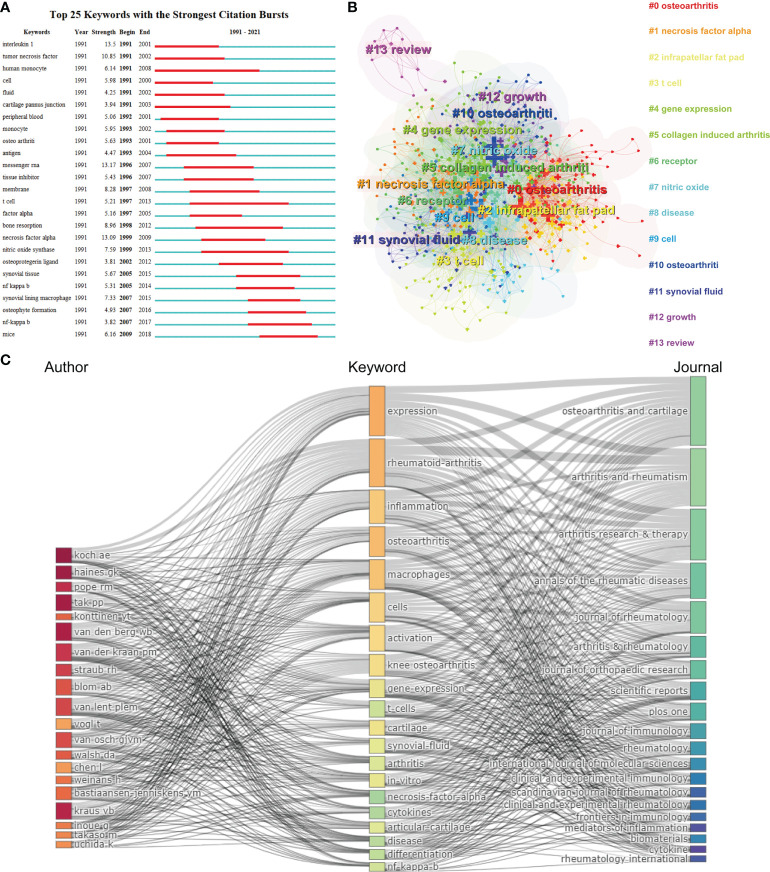
**(A)** Top 25 keywords with the strongest citation bursts based on CiteSpace. **(B)** Clustering analysis of the keyworks network based on CiteSpace. **(C)** Three-field plot of the Keywords Plus analysis on macrophages associated with osteoarthritis Notes: three-field plot of the keywords analysis: (middle field: keywords; left field: authors; right field: journals).

CiteSpace’s algorithm was also used to detect the burst of keywords based on burst detection. The top 25 keywords with the highest burst strength are shown in **Figure 7A**. We found that the keyword with highest citation outbreaks was interleukin 1 (strength = 13.5), followed by messenger RNA (13.17) and necrosis factor alpha (13.09). The keyword with the longest burst time was human monocyte, which lasted 18 years from 1991 to 2008. More meaningfully, the keyword “mice” had outbreak citations most recently (2009-2018), which implied that the research on the linkage between macrophages associated with osteoarthritis and animal models researches might be research hotspots in the future. We also built a network map to visualize keyword clusters (**Figure 7B**), and we found that “osteoarthritis” (Cluster0), “necrosis factor alpha” (Cluster1), “infrapatellar fat pad” (Cluster2), “t cell” (Cluster3), “collagen induced arthritis” (Cluster5), “nitric oxide” (Cluster7), and “synovial fluid” (Cluster11) were the hotspots of research since 1991.

**Figure 7C** represents a three-field graph in which authors, keywords, and journals were associated. It was possible to observe the links between the main elements through this three-field graph and their relationship was exhibited directly by the strength of the connection links (29). The keywords most frequently used were “expression”, “rheumatoid-arthritis” “inflammation” and “osteoarthritis”, which coincide with the keywords presented in **Figure 7B**. The author’s Koch AE, Haines GK and Pope RM are strongly connected with the keyword “expression” and “rheumatoid-arthritis” establishing the relatively strongest links. In turn, it can be found that the heaviest links were related to the *Osteoarthritis and Cartilage*. Moreover, it can be seen that the *Arthritis and Rheumatism* covered most of the papers related to the keyword “expression”, “rheumatoid-arthritis”, and “inflammation”. Therefore, this visualization suggested that rheumatoid arthritis as a kind of arthritis was relative referential for osteoarthritis research.

**Figure 8:**
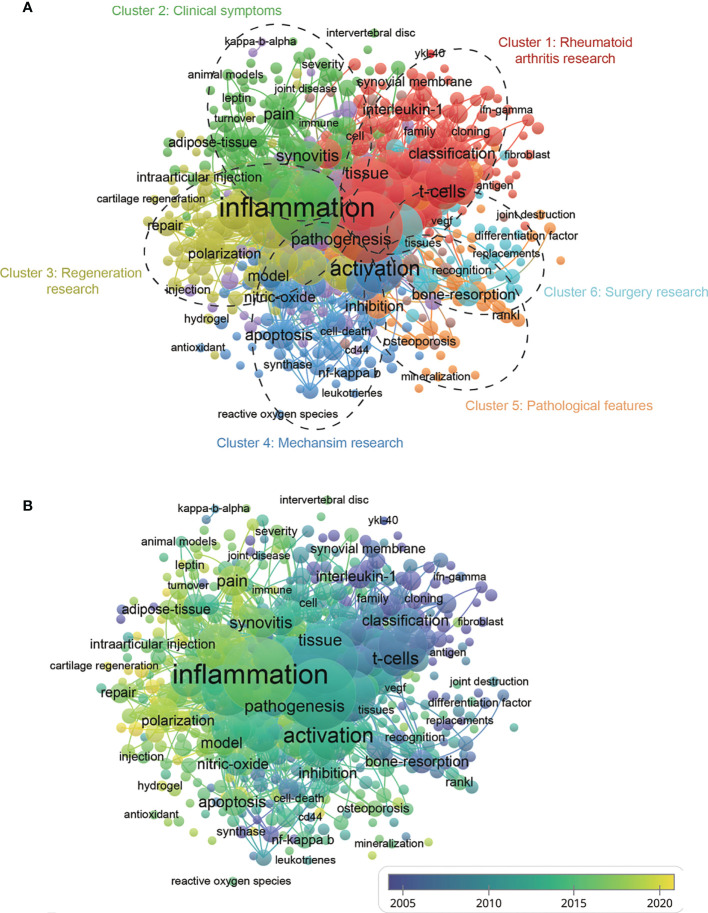
**(A)** Mapping of keywords in the research related to macrophages associated with osteoarthritis; the frequency is represented by point size and the keywords of research fields are divided into six clusters: rheumatoid arthritis research (red), clinical symptoms (green), regeneration research (yellow), mechanism (dark blue), pathological features (dark brown), and surgery research (baby blue). **(B)** Distribution of keywords according to the mean frequency of appearance; keywords in yellow appeared later than those in blue.

For bibliometrics, the keywords co-occurrence analysis is a prevalent way to identify hot research topics and areas, and it also plays a vital role in monitoring the developments in scientific research. In a co-occurrence analysis, the keyword was defined as the words used more than 5 times in titles or abstracts in all papers, which were chosen and analyzed *via* VOSviewer. As shown in **Figure 8A**, the 527 identified keywords were mainly classified into six clusters as follows: cluster 1: rheumatoid arthritis research (red), cluster 2: clinical symptoms (green), cluster 3: regeneration research (yellow), cluster 4: mechanism research (dark blue), cluster 5: pathological features (orange), and cluster 6: surgery research (light blue). These results exhibited the most prominent research topics in macrophages associated with osteoarthritis so far. In the “rheumatoid arthritis research” cluster, the primary keywords were: T cells, interleukin-1, and classification. For the “clinical symptoms” cluster, the frequently used keywords were: pain, synovitis, and adipose tissue. As for the “regeneration research” cluster, the main used keywords were: inflammation, polarization, and repair. For the “mechanism research” cluster, the dominantly used keywords were: activation, apoptosis, and nitric oxide. When talking about the “pathological features” cluster, the frequently used keywords were: inhibition, osteoporosis, and mineralization. And cluster “surgery research” consist of the frequently used keywords as follows: replacement, bone-resorption, and joint-destruction. These results exhibited that the most prominent fields of macrophages associated with osteoarthritis research included the abovementioned five directions.

According to **Figure 8B**, the VOSviewer colored all keywords based on the average times they appeared among the published papers. Specifically, the color blue indicates that the keywords appeared relatively early, while the color yellow indicates a more recent appearance. As shown in **Figure 8B**, the research trends of most studies in the six clusters were changed from rheumatoid arthritis research (cluster1), pathological features (cluster 5), and surgery research (cluster 6) to clinical symptoms (cluster 2), regeneration research (cluster 3), mechanism research (cluster 4), suggesting that future research hotspots might lie in the research of clinical symptoms, regeneration and mechanism exploration.

## Discussion

In the past few decades, researchers have put enormous efforts into macrophages associated with osteoarthritis research, and considerable progress has been achieved in diagnosing and treating osteoarthritis (30). The critical role of macrophages in inflammatory and destructive responses in OA pathogenesis is currently widely recognized. It should be noticed that increased macrophages in OA patients’ synovium and subchondral bone tissue were identified with multiple cell surface markers such as CD163, CD68, CD14, MHC class II genes and F4/80, and the increase of CD14 and CD163 is associated with OA severity (8, 31). Therefore, a significant obstacle within macrophages associated with osteoarthritis research is the development of basic studies and effective treatments.

### The trend overview of development of macrophages associated with osteoarthritis

As shown in this study, a significant increase in the number of publications per year has been found from 1 January 1991 to 31 December 2021. Moreover, the RRI has also increased slightly over the past few years, suggesting the popularity of this area is also increasing. In terms of national contributions, in our study, approximately 65 countries have published papers on the macrophages associated with osteoarthritis field. Particularly, The USA contributed the largest papers (394, 29.266%) than China (247, 17.093%), Japan (166, 11.488%), Germany (129, 8.927%), and England (115, 7.958%). Recently, the number of total citations, per citations, and H-index are critical parameters in the bibliometric study and can also show the quality and academic impact of different countries. As shown in **Figure 2** and **Figure 3**, the USA contributed the most publications, more extensive total citations, and the largest H-index, suggesting that the USA was a highly productive and leading country in this field. The USA possesses the most elite researchers and institutions worldwide, suggesting the USA’s leading position in the field of macrophages associated with osteoarthritis research. Interestingly, Scotland ranked first in terms of average citations (124.6), followed by Wales (99) and the Netherlands (73.2). Regarding the top countries or regions, it can be seen that the Netherlands, ranking sixth in the number of publications, is still making a significant progression in this field of total citation, and H-index for it ranked second and fourth, respectively. Although China ranked the second largest number of total publications, it showed weaker performance in total citations, average citations, and H-index, suggesting that China might not catch up with the USA in the following decades. The contradiction between the quantity and quality of publications in China also requires more in-depth studies. Among the scientific institutions, League of European Research Universities ranked second (127 publications), Northwestern University (39 publications), and Radboud University Nijmegen (36 publications) actively contributed to the research front. Notably, the leading top 5 institutes have contributed significantly to the research regarding with macrophages associated with osteoarthritis, which is consistent with the global publications produced by the top 5 countries. It is noted that approximately the top 25 institutes come from the top 5 countries, indicating the leading role of first-class institutes in improving one country’s academic research ranking. Therefore, this evidence collectively infers that further in-depth studies with cooperation could play a vital role in macrophages associated with osteoarthritis research, guiding researchers to publish high-quality papers in the future.

### Status and quality of authors, journals, and studies

Regarding authors, the top-ranked authors with the most publications are Americans, together with the largest funds provided by the USA National Institutes of Health (NIH), which means that the USA has played the most crucial role in the field of macrophages associated with osteoarthritis research. The top-ranked authors listed in **Table 1** with the most publications were relative earlier entrants and might have been given prior attention to obtaining the new advancements in macrophages associated with osteoarthritis research. Additionally, the collaboration analysis in **Figure 4C** showed that the research relationship among authors in different countries is relatively scattered, indicating a lack of academic connection and communication among authors. Therefore, authors in different countries and institutions should strengthen their cooperation to improve macrophages’ research on osteoarthritis jointly. As shown in **Figure 4D**, Bondeson J, Blom AB, and Goldring MB might be the top authors with the highest citation frequency, which represents the international attention and recognition of these researchers in this field.

Besides the authors’ analysis, the journals associated with publications were further explored, and the results are shown in **Table 2**. The journal *Arthritis and Rheumatism*, *Osteoarthritis and Cartilage*, and *Arthritis Research Therapy* published most papers. Recently, the impact factors were generally high. Interestingly, the top 5 journals published more than 40 papers in total, and, predictably, the listed top 10 journals might be the possible choices for researchers to publish high-quality research in the future. Furthermore, the co-citation analysis based on journals was conducted to investigate the impacts of publications by analyzing the total citation number. **Figure 5A** showed that *Osteoarthritis and Cartilage* had made the most outstanding contributions in this field. Among the top 10 research orientations, two are specialized in the clinical study and five are in basic research. More specifically, the dual-map analysis reflected the concentration of research in genetics, immunology, and rehabilitation studies.

The impact of published literature was evaluated in citation analysis of documents (**Figure 6A**) and co-citation network analysis (**Figure 6B**). **Table 4** showed that the most cited article was the exploitation of the well-characterized up-regulation of folate receptors on activated macrophages, which may be a target for rheumatoid arthritis and inflammatory osteoarthritis treatment (32). Another study focused on the role of cytokines in OA pathophysiology was written by Pelletier JP et al. (33). Among the ten most cited articles, most types of literature are of the basic research type, focusing on the pathology, pathogenesis, diagnosis, and treatment of OA and other kinds of arthritis.

Interestingly, co-citation analysis of references can figure out which publications have made the most outstanding contributions in this field. As shown in **Figure 6B**, “Differential role for interleukin-1 in induced instability osteoarthritis and spontaneously occurring osteoarthritis in mice” authored by Blom AB et al. might be the top reference with the highest citation frequency. In **Figure 6C**, most of the top 25 cited articles with the strongest citation bursts were related to OA pathophysiology, diagnosis, and therapy, indicating that these directions are hot topics in macrophages associated with osteoarthritis research field.

### Research hotspots and frontiers

The co-occurrence analysis of keywords and bursts reflected the developing trends and hotspots in macrophages associated with osteoarthritis research. As shown in **Figure 7A**, “interleukin 1” is the keyword with the highest citation outbreaks, which represents the initial status of this keyword in OA research. For example, as early as the 1990s, Arend WP et al. proposed the IL-1 receptor antagonists (IL-1Ra) intervention in the treatment of OA and confirmed a reduction of cartilage destruction associated with this therapy (34, 35). As shown in **Figures 7B, C**, it is shown that the primary research clusters mainly refer to “osteoarthritis”, “necrosis factor alpha”, “t cell”, “gene expression”, and “synovia fluid”, indicating that molecular biology exploration in OA disease is another hotspot.

In our study, the keywords’ co-occurrence network was depicted based on the determination of keywords in the titles/abstracts of all included publications. **Figure 8A** showed 6 main research trends, which could be divided into 6 clusters: rheumatoid arthritis research (red), clinical symptoms (green), regeneration research (yellow), mechanism research (dark blue), pathological features (orange) and surgery research (light blue). These results could not only comply with hopeful hotspots in this field of macrophages associated with osteoarthritis research but also forecast the directions of future studies, as follows.

(I). Rheumatoid arthritis research: Co-occurrence analysis of keywords identified “T cells”, “interleukin-1”, and “classification” as important research hotspots which deserve further attention. Rheumatoid arthritis (RA) has been considered an autoimmune disease because it presents with a chronic systemic inflammatory disorder (36). T lymphocytes (T cells), mainly categorized into helper T cells (Th cells) and cytotoxic T cells (Tc cells), secrete cytokines to modulate the behavior of cells involved in immunologic response (37). In RA, T-lymphocytes stimulate macrophages to overproduce inflammatory cytokines. Notably, the role of T cells in OA disease progression is also an emerging topic of investigation. For example, OA patients present with enhanced T helper cells in synovial tissue and synovial fluid. Furthermore, multiple T cells such Th1, Th9, and Th17 cells are located in OA synovial fluid, while Th1, Th17, and cytotoxic T cells mainly existed in OA synovial tissue, all of these cells secrete various catabolic cytokines, including IL‐2, IFN‐γ, and TNF‐α (38). Notably, the classification of osteoarthritis subtypes according to the distinct molecular signatures was performed recently. A study conducted by Yuan, Chunhui, et al. divided OA patients into four subtypes based on the symptoms: glycosaminoglycan metabolic disorder subtype, collagen metabolic disorder subtype, activated sensory neuron subtype, and inflammation subtype (39). This study provided distinct molecular subtypes in knee OA, which may shed light on the precise diagnosis and treatment of this disease.
(II). Clinical symptoms: One primary topic of OA is studying the mechanism of pain in symptomatic OA. Generally, pain is a complex process including sensory, affective, and cognitive experiences, while some kinds of tissue (infrapatellar fat pad (IFP) and the synovial membrane) have been investigated as a potential source of pain in OA (40). Regarding the role of synovitis in OA pain, Baker et al. proved the strong connection between contrast-enhanced MRI-detected synovitis and Knee OA severity (41). Another potential therapeutic target refers to adipose tissue in IFP. Hypointense IFP signal and greater volume of IFP were demonstrated to be highly correlated with OA pain (42). Specifically, the molecular mechanisms involved in OA pain refer to the IFP-Synovial membrane can be divided into neuropeptides and peptide hormones, growth factors, and cytokines (40). Interestingly, IL‐1β‐producing macrophages regulate calcitonin receptor‐like receptor (CLR) expression in synovial cells and are reported to be involved in pain transmission and neurogenic inflammation (43). In addition, the high level of Neuropeptide Y (NPY) detected in OA patients synovial fluid was also correlated with OA severity and pain (44). Both synovial fluid CD14 and CD163 were positively associated with osteophyte progression (45). Importantly, previous studies discovered that several subsets of macrophages might contribute to OA pain through nerve growth factor (NGF) and calcitonin gene-related peptide (CGRP) expression (46–48). Takano et al. discovered that CD14-positive macrophages could regulate NGF by inflammatory cytokines (IL-1β and TNF-α) production (49). In addition, Shotaro et al. reported that elevated CGRP by CD14-positive macrophages may contribute to increased OA pain (48). In addition, researchers reported that CD163+CD14^low^ macrophages expressing TNF-α might be a vital contributor to the OA pain (50). These molecular factors contribute to the pain of OA and as a potential therapeutic target in OA pain treatment and should be further explored in the future.
(III). Regeneration research: Promising regeneration strategies for OA are urgently needed since the OA involves articular cartilage destruction, synovitis, subchondral bone remodeling, osteophyte formation, and meniscus and ligament changes (5). Several specific mediators (PAMPs, DAMPs, and inflammasome) act as microenvironment stimuli that induce synovial macrophage activation and polarization (51). Since macrophage polarization plays a fundamental role in OA progression and regeneration, many efforts have been made to explore novel specific targets to inhibit or slow the progression of OA. For instance, M2 macrophage membrane-coated nanoparticles (Au-M2 NPs), a unique drug platform, could be applied as a highly anti-inflammatory and specific polarize macrophages to M2 type and eventually alleviate OA inflammation as well as matrix degradation (52). On the other hand, investigating the underlying molecular pathology of OA is also a pivotal research direction for differential treatment. For example, Yin, Jianbin, et al. performed an RNA sequencing of OA M1-polarized macrophages and successfully identified that pentraxin 3 (PTX3) is highly expressed in OA patients. Moreover, PTX3 was upregulated when miR-224-5p was insufficient, which activated the p65/NF-κB pathway to induce M1 macrophage polarization by targeting CD32 (53). Therefore, blockade of this pathway and PTX3 may alleviate the OA development.
(IV). Mechanism research: Although multiple proinflammatory factors (including IL−1, IL−6, IL−17, and TNF−α) released by chondrocytes and proliferating synoviocytes affects the mobilization, polarization and apoptosis of macrophages, the underlying mechanisms are not completely understood (54). Therefore, exploring the advanced therapeutic targets for macrophage polarization which involves OA progression, is urgently needed. Notably, nitric oxide (NO), a small bioactive molecule, can significantly inhibit the inflammatory response by activating the AMP-activated protein kinase (AMPK) signal pathway (55–57). However, the role of NO in the OA disease process remains to be elucidated; some studies suggested that NO was responsible for inducing apoptosis and proinflammatory cytokines secretion, while other studies indicated that NO and its redox derivatives might also protect chondrocytes to a certain extent (58). A study by Chen, Xu, et al. proved that A photothermal-triggered nitric oxide nanogenerator combined with siRNA attenuates macrophage-mediated inflammation, showing promising effects for OA treatment (59).
(V). Pathological features: For OA pathological progression, pathological calcification or mineralization in the affected joint is an important feature. The most common site of pathological calcification was cartilage, while other soft tissues, including the meniscus, synovium, and tendons, were also commonly affected (60). In detail, the two most common forms of pathological articular minerals refer to Basic calcium phosphate (BCP) and calcium pyrophosphate dehydrate (CPPD) (61). Several pathological processes were involved in abnormal mineralization as follows: pathological rejuvenation of chondrocytes, changes in ECM structure and composition, changes of extracellular calcium level, disordered pyrophosphate (PPi) and phosphate (Pi) metabolism, mitochondria-mediated calcification, and imbalance between inhibitors and promoters in non-collagenous proteins (NCP) (60). The relationship between osteoporosis and OA requires further investigation. In addition to commonly observed subchondral sclerosis in OA, some patients may suffer from pain and disability, thus encountering osteoporosis with increased fracture risk (62). Regarding the current situation of OA study, we suggest future research should focus on conducting more systematic prospective studies to comprehensively understand the OA pathological features.
(VI). Surgery research: The surgical indication is pivotal for OA patients because surgery is always a relative indication. Multiple indications include symptoms, OA stage, and individual patient factors (age, physical activity, and patient’s comorbidities) that should be taken into consideration in surgical interventions (63). The surgical treatment for OA main refers to arthroscopic lavage and debridement, cartilage repair techniques, osteotomies around the knee, and joint arthroplasty (63). For joint arthroplasty, it is vital to determine appropriate OA progression time points for joint replacement. Biomarkers in plasma or other body fluids could be an ideal indicator for diagnosis and determination of OA progression. For example, the CRTAC1 protein in plasma was found to be associated with joint pain and hand OA severity, and it is not associated with other inflammatory joint diseases such as rheumatoid arthritis (64). In addition, after joint replacement surgery, the protein profile in plasma also changed, indicating that these biomarkers can be used to predict prosthesis survival time or early prosthesis failure.

### Future research trends

According to the analysis above, it is significant to predict the future trends and possible future impact on search of macrophages associated with osteoarthritis. As depicted in **Figure 7**, the primary research clusters mainly refer to “osteoarthritis”, “necrosis factor alpha”, “T cell”, “gene expression”, and “synovia fluid”, indicating that molecular biology exploration in OA disease is another hotspot and future direction. In addition, as shown in **Figure 8**, the research directions have changed from rheumatoid arthritis research, pathological features, and surgery research to clinical symptoms, regeneration research, mechanism research, which could significantly influence future researchers. In terms of clinical symptoms research, many key molecules associated with OA have been identified and the relationship between subsets of macrophages and OA clinical symptoms has also been discussed, which could assist clinicians to better manage patients’ symptoms. As for the regeneration research, many researchers dedicated to explore specific targets to slow down or inhibit the progression of OA by targeting M1 or M2 macrophages. In addition, the mechanism research of macrophages has also drawn many researchers’ attention. For example, NO was found to induce apoptosis and proinflammatory cytokines secretion, while others reported that it could protect chondrocytes and attenuates macrophage-mediated inflammation (49–53). Therefore, exploring the mechanisms underlying on the macrophage and OA progression. Based on these findings, the development of basic research of molecular biology and mechanism exploration could benefit the relief of clinical symptoms.

### Limitation

There are still some limitations to be discussed: (1) Due to the limitation of our bibliometric software, all of the studies collected from WoSCC, PubMed, Cochrane, Scopus and Embase library databases have not been included, which may lead to publication bias. Therefore, more data sources and powerful software are recommended in the future research. (2) We only extracted research and review articles in English, and the articles published in non-English language or non-research/review articles were not included in this study, which may result in some omissions. (3) We did not visualize the keywords with a timeline, which may result in hotspot prediction bias due to neglection of temporal data. (4) Since the new studies are updated daily, we might neglect some influential newly published studies. (5) As the data selection is done by two authors, encountered problems were resolved by consulting with experts to reach the final consensus.

## Conclusion

In conclusion, this study is the first bibliometric analysis to scientifically and comprehensively analyze the global macrophages associated with osteoarthritis research trends over the past 30 years. This study systematically summarized the global publication trends and helped scholars identify the essential authors, institutions, and journals in this field. Moreover, the keyword and co-citation clustering analysis also guide researchers to choose new research directions mainly in five directions as follows “rheumatoid arthritis research”, “clinical symptoms”, “regeneration research”, “mechanism research”, “pathological features”, and “surgery research”. We can expect that further cooperation among authors, institutions, and countries in the future would accelerate the development of macrophages associated with osteoarthritis research.

## Data availability statement

The original contributions presented in the study are included in the article/supplementary material. Further inquiries can be directed to the corresponding authors.

## Author contributions

DX, JHL contributed to conception and design of the study. ZY, ZH, HaL organized the database. ZY, ZH, HaL organized the database. ZY, JJL, LL and HuL performed the statistical analysis. YZ, HaL wrote the first draft of the manuscript. JJL, DX, and JHL wrote sections of the manuscript. JJL and HuL contributed to data acquisition. All authors contributed to the article and approved the submitted version.

## Funding

This work was supported by Beijing Natural Science Foundation (7214261), Peking University Medicine Fund of Fostering Young Scholars' Scientific & Technological Innovation (BMU2022PYB004) and Peking University People’s Hospital Scientific Research Development Funds (RDY2020-9).

## Conflict of interest

The authors declare that the research was conducted in the absence of any commercial or financial relationships that could be construed as a potential conflict of interest.

## Publisher’s note

All claims expressed in this article are solely those of the authors and do not necessarily represent those of their affiliated organizations, or those of the publisher, the editors and the reviewers. Any product that may be evaluated in this article, or claim that may be made by its manufacturer, is not guaranteed or endorsed by the publisher.
